# Supplementary material for: The mutational landscape of a prion-like domain
Source: Nat Commun. 2019 Sep 13;10:4162. doi: 10.1038/s41467-019-12101-z (PMC6744496; doi:10.1038/s41467-019-12101-z)
Supplement: Supplementary file 1 — Supplementary Information [file 41467_2019_12101_MOESM1_ESM.pdf]

Supplementary Information

The Mutational Landscape of a Prion-like Domain

Bolognesi, Faure *et al.*

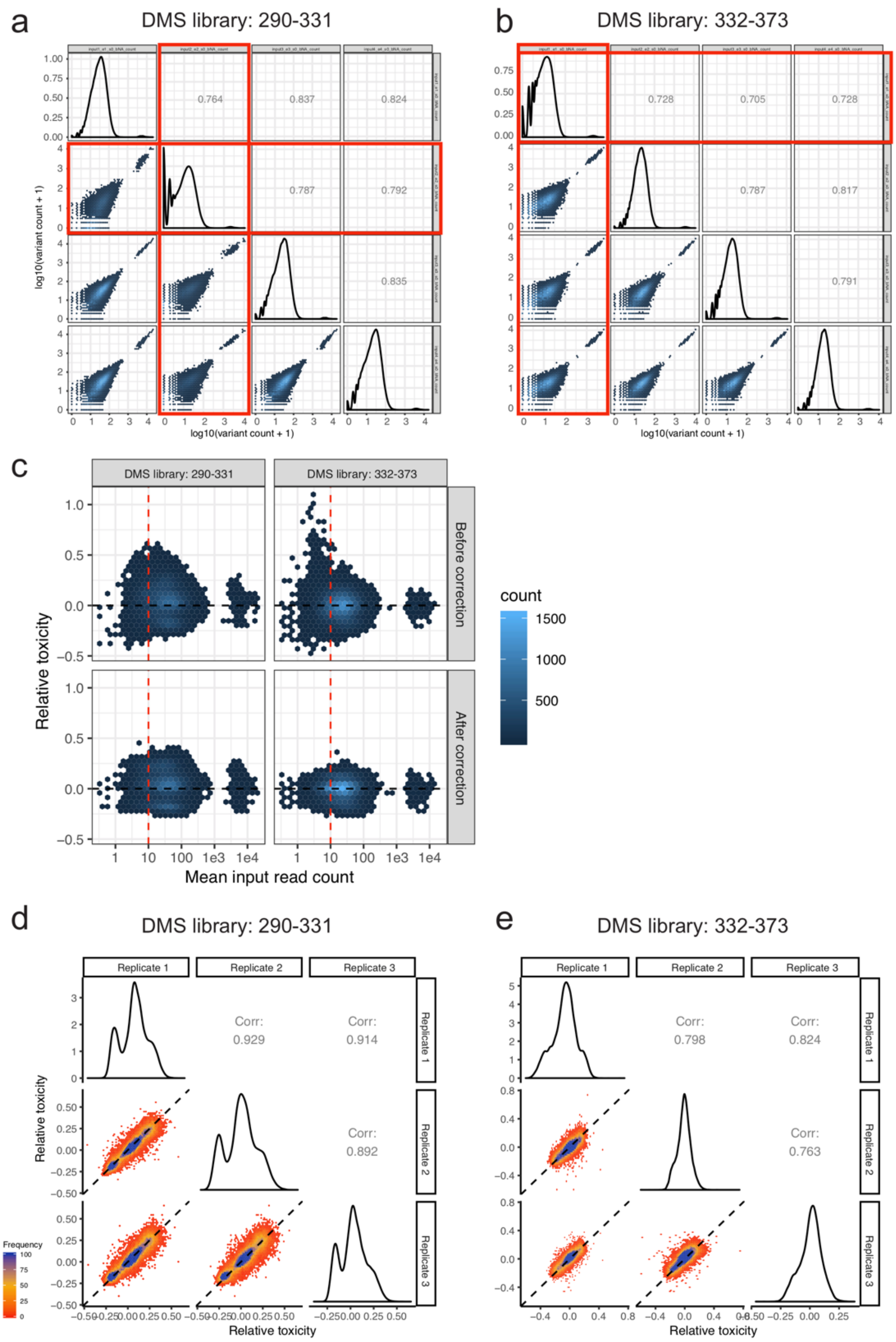

Supplementary Figure 1

Quality control and pre-processing of DMS datasets

a Correlation of mutant variant counts between all input replicates in DMS library 290-331. The Pearson correlation coefficients (R) are indicated in the upper matrix triangle. Replicate selection 2 was removed from downstream analyses due to lower than average correlation with other replicates **b** Correlation of mutant variant counts between all input replicates in DMS library 332-373. Replicate selection 1 was removed from downstream analyses due to lower than average correlation with other replicates. **c** Comparison of relative toxicity of single and double AA mutants and mean input read count for each DMS library before and after Bayesian correction of double AA mutant toxicity estimates. The vertical dashed line indicates the minimum mean input read count threshold (10) for variants used in downstream analyses. **d** Correlation of toxicity estimates between all retained replicates for single and double amino acid (AA) mutants from library 290-331. The Pearson correlation coefficients (Corr) are indicated. **e** Similar to panel d except showing results corresponding to retained replicates from library 331-373.

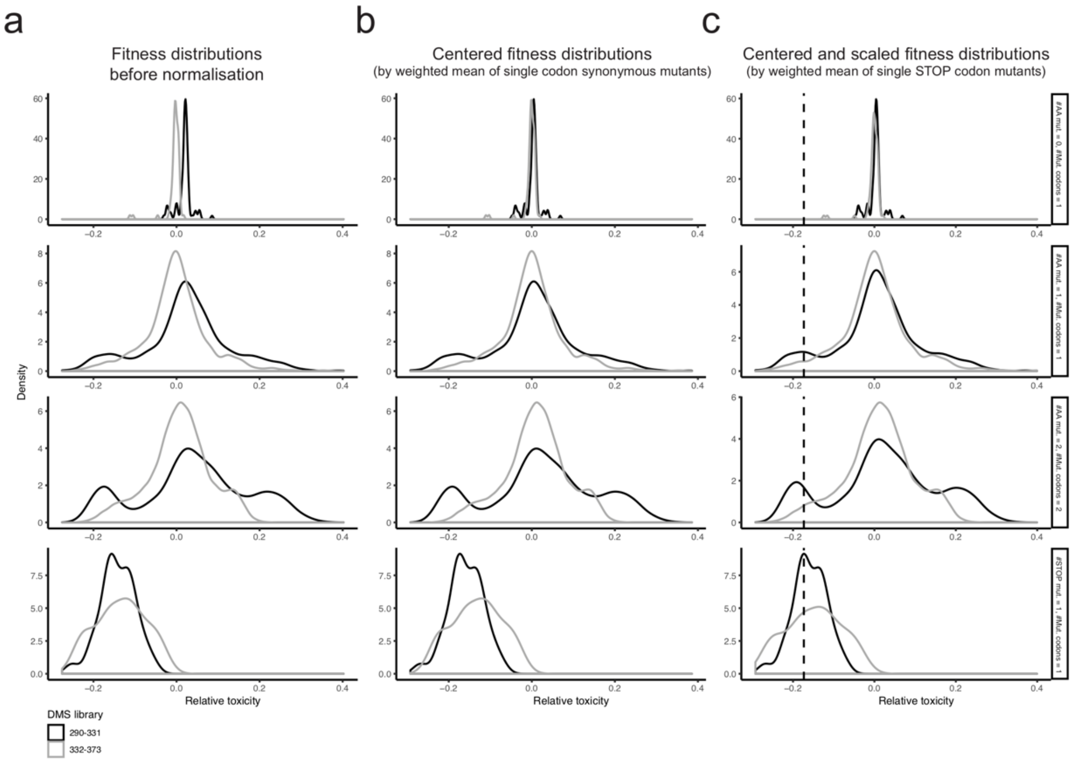

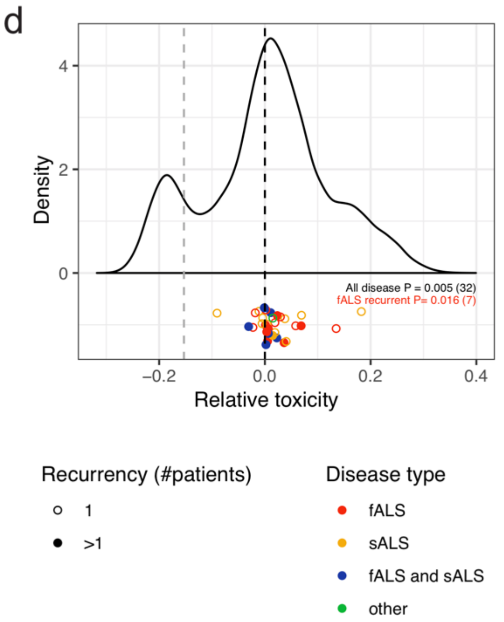

e

Name	Position	#fALS	#sALS	Reference
Gly290Ala	290	2		Van Deerlin (2008) Lancet Neurol 7, 409
Ser292Asn	292	1	2	Xiong (2010) BMC Med Genet 11, 8
Gly294Ala	294		1	Sreedharan (2008) Science 319, 1668
Gly294Val	294	4	7	Corrado (2009) Hum Mutat 30, 688
Gly295Cys	295	1		van Blitterswijk (2012) Hum Mol Genet 21, 3776
Gly295Ser	295	2	4	Corrado (2009) Hum Mutat 30, 688
Gly295Arg	295		1	Corrado (2009) Hum Mutat 30, 688
Gly298Ser	298	2		Van Deerlin (2008) Lancet Neurol 7, 409
Gln303His	303		1	Lattante (2012) Neurology 79, 66
Met311Val	311	1		Lemmens (2009) J Neurol Neurosurg Psychiatry 80, 354
Ala315Thr	315	3		Gitcho (2008) Ann Neurol 63, 535
Ala315Glu	315	1		Fujita (2011) Neurology 77, 1427
Ala321Val	321		1	Kirby (2010) Neurogenetics 11, 217
Ala321Gly	321		1	Bäumer (2009) J Neurol Neurosurg Psychiatry 80, 1283
Gln331Lys	331		1	Sreedharan (2008) Science 319, 1668
Ser332Asn	332	1		Corrado (2009) Hum Mutat 30, 688
Gly335Asp	335		1	Corrado (2009) Hum Mutat 30, 688
Met337Val	337	7		Sreedharan (2008) Science 319, 1668
Gln343Arg	343	1		Rutherford (2008) PLoS Genet 4, e1000193
Asn345Lys	345	2		Rutherford (2008) PLoS Genet 4, e1000193
Gly348Cys	348	5	4	Kabashi (2008) Nat Genet 40, 572
Gly348Val	348	2	2	Kirby (2010) Neurogenetics 11, 217
Gly348Arg	348	1		Chiang, C. et al., Sci. Rep. 6, 21581, 2016
Asn352Ser	352	8	4	Kühnlein (2008) Arch Neurol 65, 1185
Asn352Thr	352	2		Ticozzi (2011) Neurobiol Aging 32(11):2096-9
Gly357Ser	357		2	Iida (2012) Neurobiol Aging 33, 786
Gly357Arg	357	1		Chiang (2012) J Hum Genet 57, 316
Met359Val	359			Borroni (2010) Rejuvenation Res 13(5):509-17
Arg361Ser	361		1	Kabashi (2008) Nat Genet 40, 572
Arg361Thr	361	2		Chiang (2012) J Hum Genet 57, 316
Pro363Ala	363		1	Daoud (2009) J Med Genet 46, 112
Gly368Ser	368		1	De Marco (2011) Acta Neuropathol 121, 611

Supplementary Figure 2

Inter-library normalisation of DMS datasets and toxicity of human disease mutations

a Toxicity distributions of single codon synonymous (silent) variants (top), single and double mutants (middle) and single STOP codon variants, shown separately for each library (see colour key) and before centering and scaling. **b** As in panel a, but after inter-library normalization by centering on the error-weighted means of toxicity of single codon synonymous (silent) variants. **c** As in panel b, but after additionally scaling such that the error-weighted means of single STOP codon variants coincided. Mean toxicity of variants with single STOP codon mutation is indicated by dashed vertical line. **d** Relative toxicities of classified human disease AA substitution variants (coloured dots; see key) below the relative toxicity distribution of all single and double AA mutants assayed in this study. Disease variants observed in more than one patient are classified as recurrent. **e** Table of all human disease AA substitution mutations in TDP-43 used in panel d.

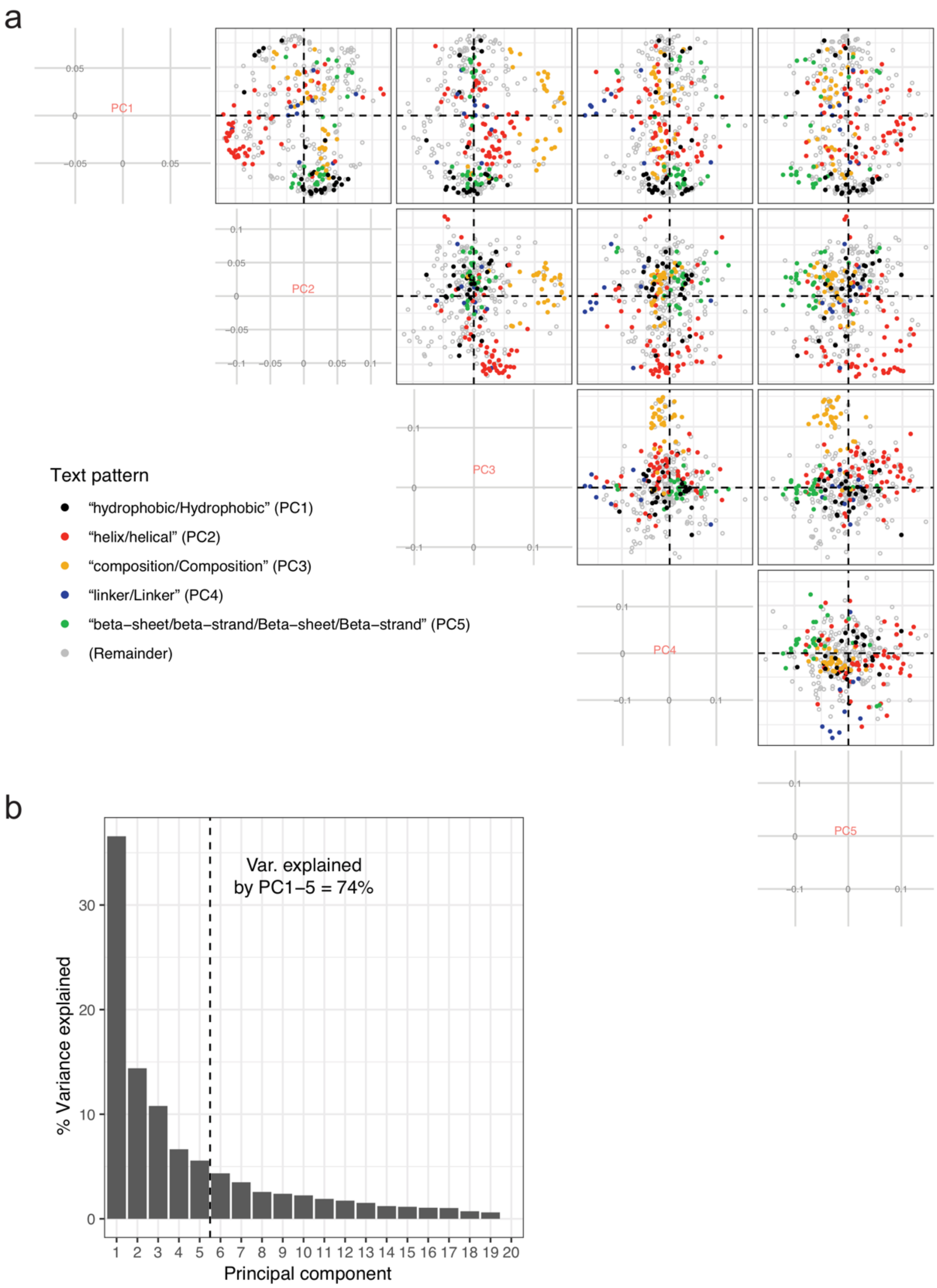

Supplementary Figure 3

Principle components analysis of amino acid physicochemical properties

a Results of PCA of a curated collection of numerical indices representing various physicochemical and biochemical properties of AAs (see Methods). Biplot matrix indicating variable loadings of the top 5 PCs. Colours indicate text matches to index descriptions (see colour key). **b** Screeplot indicating percentage variance explained by all PCs.

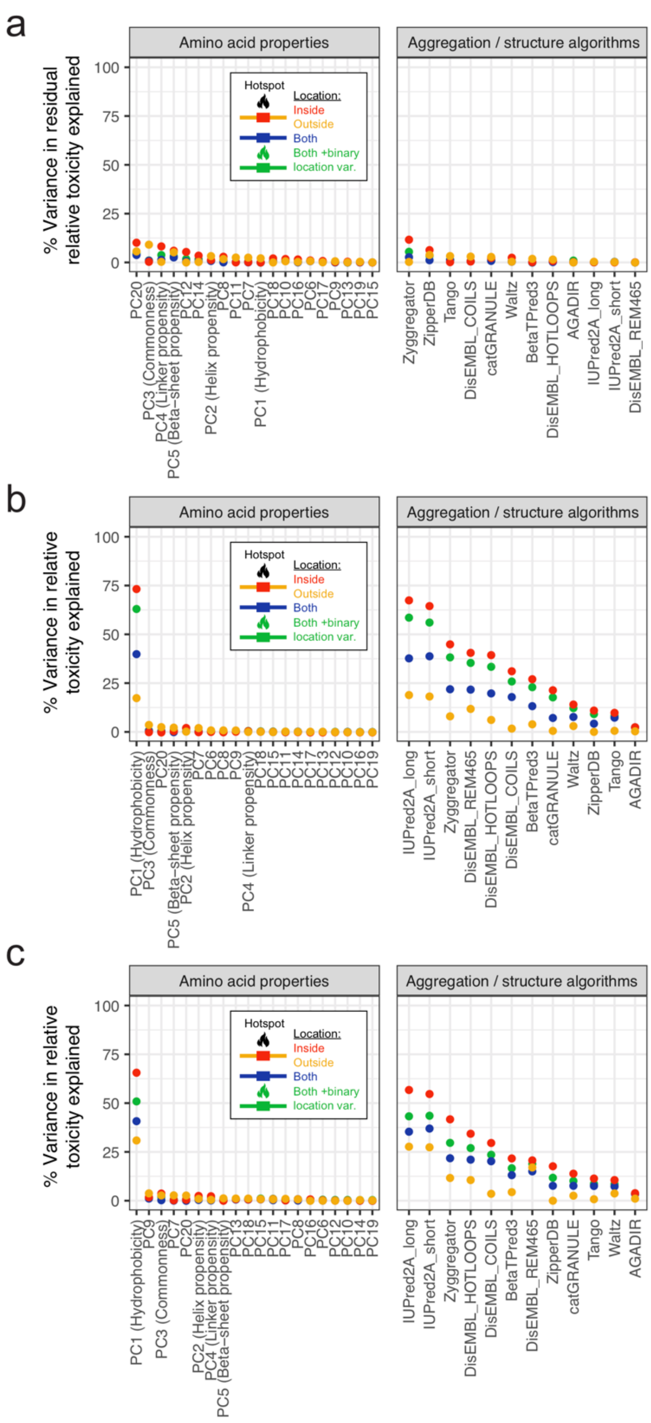

Supplementary Figure 4

Linear regression models to predict mutant variant toxicity

a Percentage variance of residual relative toxicity (after controlling for hydrophobicity and location) explained by linear regression models predicting single and double mutant variant toxicity from changes in AA properties upon mutation (left) and using scores from aggregation/structure algorithms (right). Different regression models were built for different subsets of the data. Simple linear regression models for all variants (blue) or only variants inside (red) or outside (yellow) the hotspot region. And a regression model using all variants and including a binary location variable (inside/outside hotspot) as well as an interaction term between binary location variable and the indicated AA property feature (green). **b** Percentage variance of relative toxicity explained by linear regression models predicting single mutant variant toxicity from changes in AA properties upon mutation (left) and using scores from aggregation/structure algorithms (right). **c** Similar to panel b except showing results using double mutant variants.

a

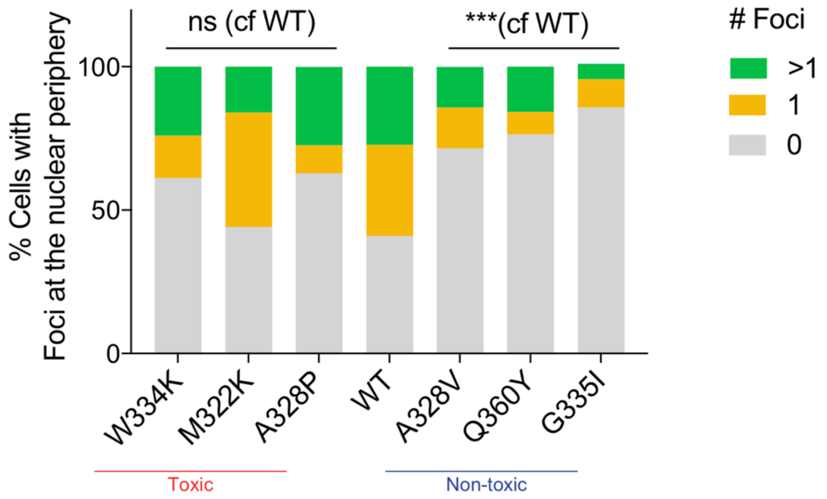

b

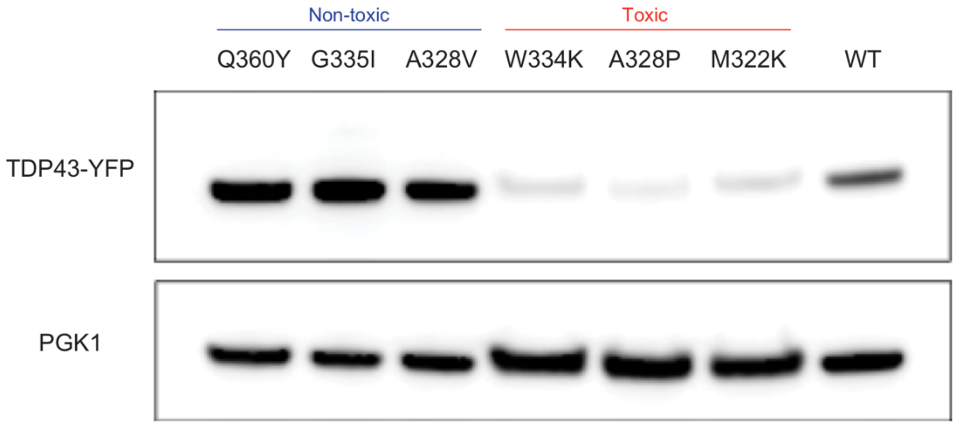

Supplementary Figure 5

Scoring of intracellular phenotypes and expression of toxic and non-toxic mutants

a Scoring of intracellular phenotypes by automated foci counting**.** Percentage of cells with foci at the nuclear periphery automatically scored by CellProfiler. Fisher's Exact test. **b** Immunohistochemistry of toxic and non-toxic mutants. Expression of different TDP-43 variants after 8h induction of protein expression in Galactose was measured by Western Blotting. Phosphoglycerate Kinase 1 was used as a Loading Control. Source data are provided as a Source Data file.

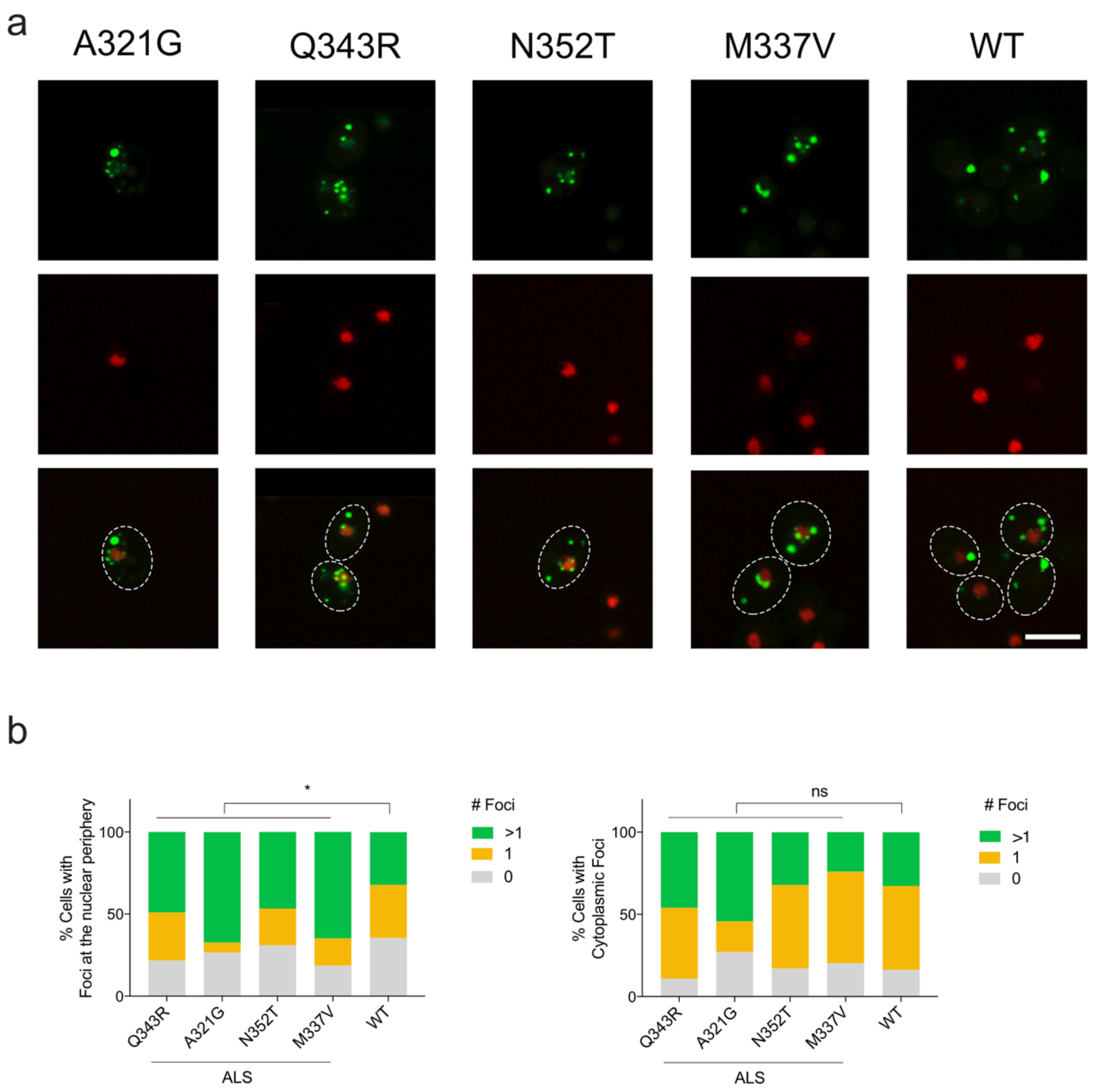

Supplementary Figure 6

Scoring of intracellular phenotypes for ALS mutants

a Representative fluorescence microscopy Images of yeast cells expressing indicated YFP-tagged TDP-43 variants (green). H4-mCherry marks nuclei (red). **b** Percentage of cells with foci at the nuclear periphery (left) or cells with cytoplasmic foci (right). Cells scored: n[disease variants]=403, n[WT]=61. Fisher's exact test. Scale bar = 5 μM. Source data are provided as a Source Data file.

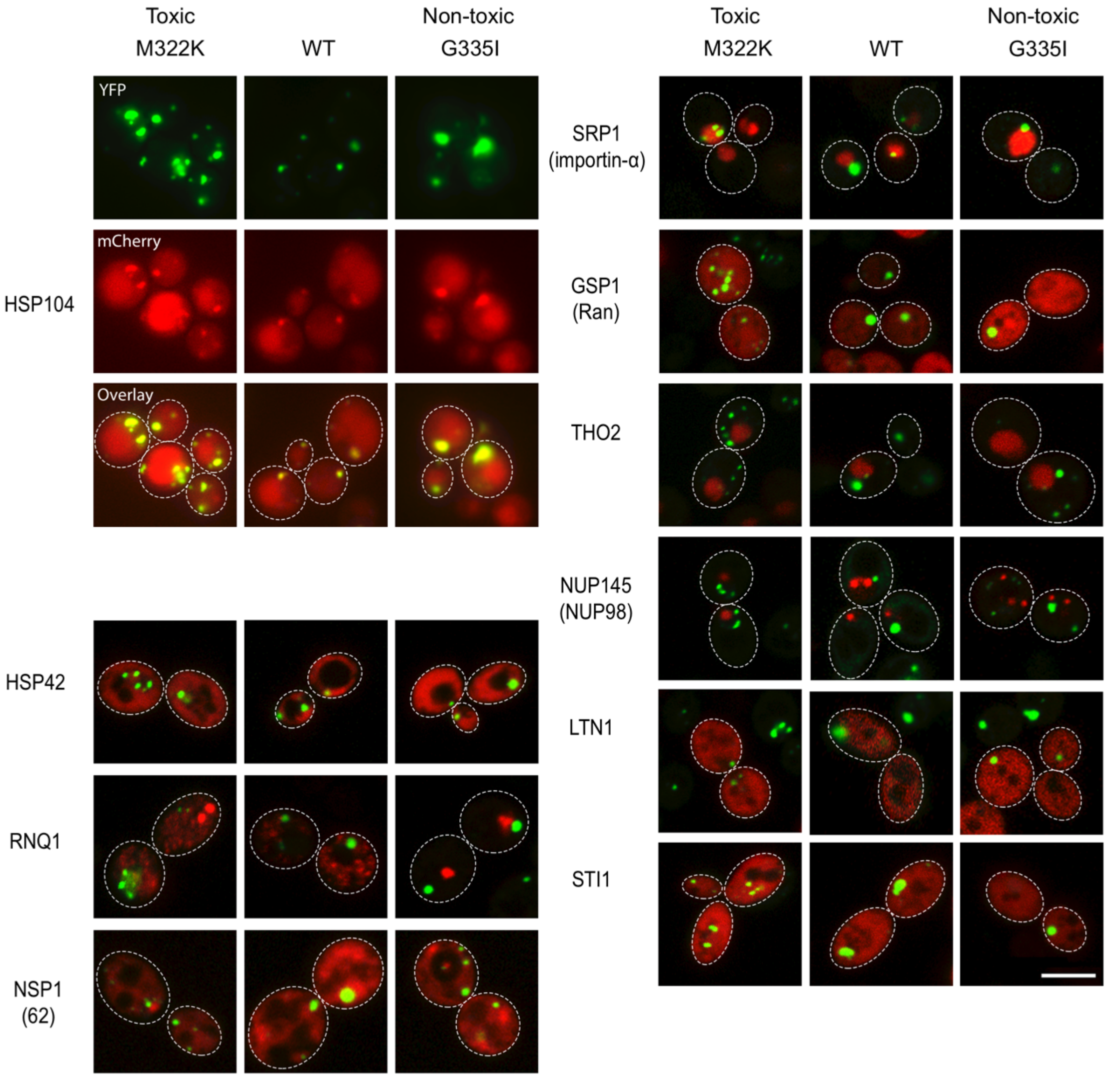

Supplementary Figure 7

Co-localization with known IPOD, JUNQ and Nuclear Pore markers

Representative fluorescence microscopy images of yeast cells expressing indicated YFP-tagged TDP-43 variants (green) and mCherry-tagged proteins (red). Markers and regulators of the insoluble protein compartment (IPOD) or the juxtanuclear quality control compartment (JUNQ): Hsp104, Hsp42, Rnq1, Ltn1, Sti1[1,2] Nuclear pore elements: Nup145, Gsp1, Tho2, Srp1, Nsp1[3,4,5] . The human homologues are highlighted in brackets.

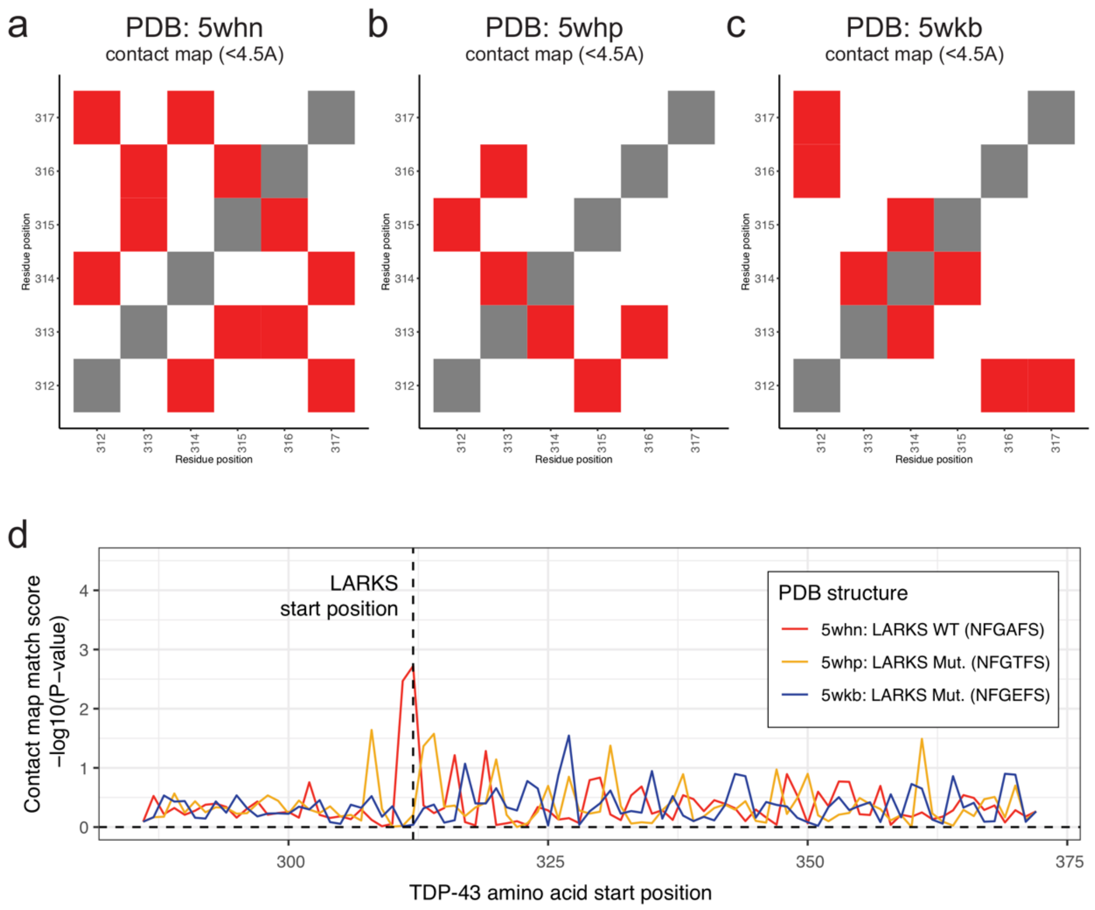

Supplementary Figure 8

LARKS structure propensities

a Contact matrix based on a minimal side-chain heavy atom distance of 4.5Å derived from WT LARKS PDB structure 5whn. **b** Contact matrix derived from mutant LARKS PDB structure 5whp. **c** Contact matrix derived from mutant LARKS PDB structure 5wkb. **d** LARKS structure propensities for PDB-structure derived contact matrices shown in panels a-c (see Methods). The dashed vertical line indicates the start position of the LARKS (TDP-43 AA residue 312).

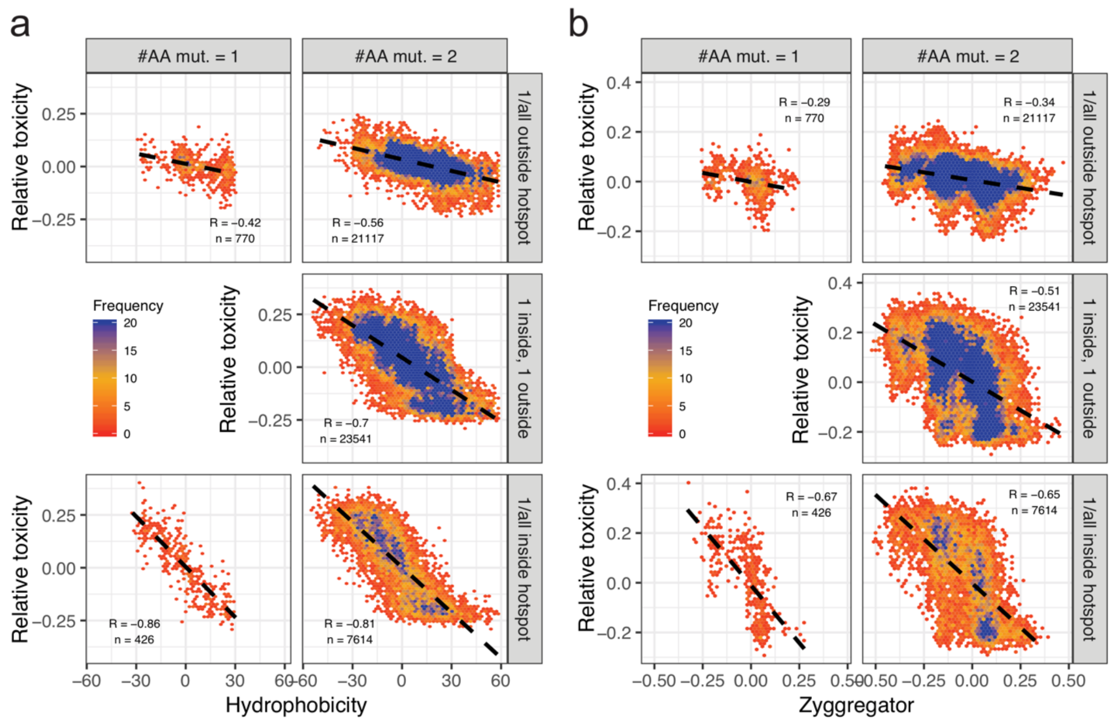

Supplementary Figure 9

Toxicity of variants as a function of hydrophobicity and aggregation propensity

a Toxicity of variants with single (left) or double (right) mutations occurring outside (top), inside (bottom) or in both locations (1 outside/1 inside) w.r.t. the hotspot region (middle), as a function of hydrophobicity changes (PC1) induced by mutation. The Pearson correlations (R) before binning are indicated. **b** Toxicity of variants with single (left) or double (right) mutations occurring outside (top), inside (bottom) or in both locations (1 outside/1 inside) w.r.t. the hotspot region (middle), as a function of changes in aggregation propensity (Zyggregator). The Pearson correlations (R) before binning are indicated.

Supplementary References

1. D. Kaganovich, R. Kopito, J. Frydman, Misfolded proteins partition between two distinct quality control compartments. *Nature*. **454**, 1088–1095 (2008).

2. S. M. Hill, S. Hanzén, T. Nyström, Restricted access: spatial sequestration of damaged proteins during stress and aging. *EMBO Rep.* **18**, 377–391 (2017).

3. A. C. Woerner *et al.*, Cytoplasmic protein aggregates interfere with nucleocytoplasmic transport of protein and RNA. *Science*. **351**, 173–176 (2016).

4. F. Gasset-Rosa *et al.*, Cytoplasmic TDP-43 De-mixing Independent of Stress Granules Drives Inhibition of Nuclear Import, Loss of Nuclear TDP-43, and Cell Death. *Neuron* (2019), doi:10.1016/j.neuron.2019.02.038.

5. C.-C. Chou *et al.*, TDP-43 pathology disrupts nuclear pore complexes and nucleocytoplasmic transport in ALS/FTD. *Nat. Neurosci.* (2018), doi:10.1038/s41593-017-0047-3.
