# Supplementary material for: The mutational landscape of a prion-like domain
Source: Nat Commun. 2019 Sep 13;10:4162. doi: 10.1038/s41467-019-12101-z (PMC6744496; doi:10.1038/s41467-019-12101-z)
Supplement: Supplementary file 3 — Reporting Summary [file 41467_2019_12101_MOESM3_ESM.pdf]

nature research

Corresponding author(s): Ben Lehner, Benedetta Bolognesi

Last updated by author(s): Aug 14, 2019

Reporting Summary

Nature Research wishes to improve the reproducibility of the work that we publish. This form provides structure for consistency and transparency in reporting. For further information on Nature Research policies, see Authors & Referees and the Editorial Policy Checklist.

Statistics

For all statistical analyses, confirm that the following items are present in the figure legend, table legend, main text, or Methods section.

n/a	Confirmed	
☐	☒	The exact sample size (*n*) for each experimental group/condition, given as a discrete number and unit of measurement
☐	☒	A statement on whether measurements were taken from distinct samples or whether the same sample was measured repeatedly
☐	☒	The statistical test(s) used AND whether they are one- or two-sided<br>*Only common tests should be described solely by name; describe more complex techniques in the Methods section.*
☐	☒	A description of all covariates tested
☐	☒	A description of any assumptions or corrections, such as tests of normality and adjustment for multiple comparisons
☐	☒	A full description of the statistical parameters including central tendency (e.g. means) or other basic estimates (e.g. regression coefficient) AND variation (e.g. standard deviation) or associated estimates of uncertainty (e.g. confidence intervals)
☐	☒	For null hypothesis testing, the test statistic (e.g. *F*, *t*, *r*) with confidence intervals, effect sizes, degrees of freedom and *P* value noted<br>*Give P values as exact values whenever suitable.*
☒	☐	For Bayesian analysis, information on the choice of priors and Markov chain Monte Carlo settings
☒	☐	For hierarchical and complex designs, identification of the appropriate level for tests and full reporting of outcomes
☐	☒	Estimates of effect sizes (e.g. Cohen's *d*, Pearson's *r*), indicating how they were calculated

Our web collection on statistics for biologists contains articles on many of the points above.

Software and code

Policy information about availability of computer code

Data collection	LAS AF software was used for all imaging.
Data analysis	All software code and custom scripts are available on GitHub: https://github.com/lehner-lab/DiMSum for raw read processing, https://github.com/lehner-lab/tardbpdms for all downstream analyses and to produce all figures, and https://github.com/lehner-lab/tardbpdms_cellprofiler_scripts for CellProfiler pipelines.

For manuscripts utilizing custom algorithms or software that are central to the research but not yet described in published literature, software must be made available to editors/reviewers. We strongly encourage code deposition in a community repository (e.g. GitHub). See the Nature Research guidelines for submitting code & software for further information.

Data

Policy information about availability of data

All manuscripts must include a data availability statement. This statement should provide the following information, where applicable:

- Accession codes, unique identifiers, or web links for publicly available datasets
- A list of figures that have associated raw data
- A description of any restrictions on data availability

Raw sequencing data and the processed data table (Supplementary Table 3) have been deposited in NCBI's Gene Expression Omnibus and are accessible through the GEO Series accession number GSE128165 (https://www.ncbi.nlm.nih.gov/geo/query/acc.cgi?acc=GSE128165).

Field-specific reporting

Please select the one below that is the best fit for your research. If you are not sure, read the appropriate sections before making your selection.

☒ Life sciences ☐ Behavioural & social sciences ☐ Ecological, evolutionary & environmental sciences

For a reference copy of the document with all sections, see nature.com/documents/nr-reporting-summary-flat.pdf

Life sciences study design

All studies must disclose on these points even when the disclosure is negative.

Sample size	Sample size for the selection experiments was determined on the basis of the number of variants in each library (378 all single nt mutations and 70875 all double nt mutations).<br>No sample-size calculations were performed for the cell biology experiments. Sample size was determined to be adequate based on the magnitude and consistency of statistically tested differences between groups.
Data exclusions	One out of four input replicates (and all associated output samples) was discarded due to considerably lower correlations with the other replicates . Some FRAP traces were excluded as they were too noisy to be fitted accurately
Replication	Selection experiments were performed in 4 independent biological replicates (more details in the methods Section). Number of replicates for other experiments are described in each figure legend.
Randomization	*Describe how samples/organisms/participants were allocated into experimental groups. If allocation was not random, describe how covariates were controlled OR if this is not relevant to your study, explain why.*
Blinding	*Describe whether the investigators were blinded to group allocation during data collection and/or analysis. If blinding was not possible, describe why OR explain why blinding was not relevant to your study.*

Reporting for specific materials, systems and methods

We require information from authors about some types of materials, experimental systems and methods used in many studies. Here, indicate whether each material, system or method listed is relevant to your study. If you are not sure if a list item applies to your research, read the appropriate section before selecting a response.

Materials & experimental systems

n/a	Involved in the study	
☐	☒	Antibodies
☒	☐	Eukaryotic cell lines
☒	☐	Palaeontology
☒	☐	Animals and other organisms
☒	☐	Human research participants
☒	☐	Clinical data

Methods

n/a	Involved in the study	
☒	☐	ChIP-seq
☒	☐	Flow cytometry
☒	☐	MRI-based neuroimaging

Antibodies

Antibodies used	anti-GFP mouse antibody (Santa Cruz sc-9996) , anti-PGKD1 mouse antibody (Novex 459250)
Validation	*Describe the validation of each primary antibody for the species and application, noting any validation statements on the manufacturer's website, relevant citations, antibody profiles in online databases, or data provided in the manuscript.*
