# Supplementary material for: The mutational landscape of a prion-like domain
Source: Nat Commun. 2019 Sep 13;10:4162. doi: 10.1038/s41467-019-12101-z (PMC6744496; doi:10.1038/s41467-019-12101-z)
Supplement: Supplementary file 4 — Description of Additional Supplementary Files [file 41467_2019_12101_MOESM4_ESM.docx]

**Description of Additional Supplementary Files**

File Name: Supplementary Data 1
Description: DNA Sequence of Primers

File Name: Supplementary Data 2
Description: Curated collection of numerical indices representing various physicochemical and biochemical properties of amino acids

File Name: Supplementary Data 3
Description: Merged and normalised toxicity estimates

File Name: Supplementary Data 4
Description: Toxicity estimates from independent replicates before merging and normalisation
