# Supplementary material for: The mutational landscape of a prion-like domain
Source: Nat Commun. 2019 Sep 13;10:4162. doi: 10.1038/s41467-019-12101-z (PMC6744496; doi:10.1038/s41467-019-12101-z)
Supplement: Supplementary file 2 — Peer Review File [file 41467_2019_12101_MOESM2_ESM.pdf]

Reviewers' comments:

Reviewer #1 (Remarks to the Author):

In this manuscript Bolognesi, Faure, et al. use deep mutational scanning to study the impact of protein aggregation on cellular toxicity. They introduce more than fifty thousand mutations in the prion-like domain (PrD) of the intrinsically disordered human protein TDP-43 and quantified their cytotoxicity in yeast. They find that mutations increasing hydrophobicity and aggregation tend to reduce toxicity, consistent with models suggesting that mature protein aggregates are not themselves toxic. The authors propose a mechanism in which larger, solid protein aggregates titrate protein away from smaller and dynamic liquid-like condensates in the nuclear periphery. Presumably, these liquid-like protein condensates may interfere with protein transport across the nuclear membrane. The mutations with the largest effect on toxicity tend to cluster in a central hotspot withinin the PrD. The authors developed a clever computational method based on epistatic interactions that points to the in vivo secondary structure of the protein (Nature Genetics, in press) andused it in this study to show that this central hotspot, rather than being disordered, is indeed structured inside yeast cells.

This paper adds significant evidence to the body of work suggesting that protein aggregates associated with neurodegenerative diseases, in contrast to prevailing opinion, may ameliorate rather than enhance cell toxicity. This is a solid piece of work. The results are very interesting and convincing and, in my view the claims made are supported by the evidence. In my view it should be published without delay.

Minor comments:

1. Some additional introduction of the concept of liquid demixing would be helpful for many readers.

2. It seems to me that dosage-sensitive proteins may also lead to a fitness cost when concentration decreases below threshold. Could the authors comment?

3. Why do the non-toxic protein variants had higher concentration as quantified by Western?

4. How can we know that aggregation potential accounts for 4% of the variance on relative toxicity in the hotspot from Fig. 2F,G? Is this simply because of the R? It would be good to add a sentence or two explaining this.

5. In my view Panel 2C does not show that "None of them are as predictive as hydrophobicity"

Reviewer #2 (Remarks to the Author):

To explore the relationship between TDP-43 aggregation and toxicity, here the authors used deep mutagenesis, generating >50,000 mutations comprising of 1266 single and 56, 730 double mutants, in the PRD of TDP-43 and quantifying toxicity in yeast. Of these mutants, 18032 were more toxic than WT TDP-43 and 16152 less toxic. The authors found that mutations that increased the aggregation propensity of TDP-43 correlated with reduced toxicity, whereas those that promoted dynamic, liquid condensates correlated with increased toxicity. Interestingly, increasing the hydrophobicity of TDP-43, which is associated with reduced toxicity, caused a relocalization of the protein away from the nuclear membrane and formation of larger aggregates. Mutational effect was strongest in a 31 aa hotspot at residues 312-342 with mutations to charged or polar residues increasing toxicity and to hydrophobic aa decreasing toxicity. From these findings, the authors propose that TDP-43 aggregation is neuroprotective by titrating from liquid-like states.

This is an interesting observation. Comm ents are below

1. What was the finding from disease causing mutations e.g. A315T, M337V etc ?

2. WT TDP-43 should be included in Fig 3A

3. Further characterization of the different types of TDP-43 inclusions is required e.g. are the aggregates p62 positive, do the droplets co-localize with stress granule markers, nuclear pore proteins?

Reviewer #3 (Remarks to the Author):

The manuscript by Bolognesi et al. describes deep mutational scanning results of TDP-43 toxicity in yeast. This review is focused on the deep mutational scanning approach and data. The authors purchased about 150 based synthetic oligos doped with random mutations at a frequency intended to yield a ratio of about 1:2:2 of WT: single mutation: double mutation. 2 libraries of mutants were cloned into galactose inducible plasmids, introduced into budding yeast, and subject to bulk growth competitions under induced conditions. The frequency of each mutant was estimated by paired-end sequencing of samples prior to and after competition. The change in frequency of each mutated variant was used to estimate impacts on growth rate.

Many aspects of the mutational scanning data appear to have been done with careful thought. However, there are also aspects of the mutational scanning data that are not clearly described or that may be potentially misleading and should be addressed prior to publication. The correlation between replicates (Fig 1B) is impressive and indicates that stochastic noise to signal is favorable.

Major concerns:

1. The treatment of biological replicates is unclear. Four biological replicates are mentioned in the method section, but two replicates are presented in Fig 1B, and one set of measurements is provided in Sup. Table S3. The authors need to clarify how replicates were performed and analyzed. Each replicate needs to be reported in a separate Supplementary Table. This is imperative not only for judging the merit of the data, but also for potential future analyses to distinguish biological from technical variation.

2. Misreads need to be experimentally examined. Assuming that paired-end reads eliminate mis-reads is unwise. Potential misreads should be assessed using a homogenous starting sequence (e.g. wild-type plasmid) and the same procedure for preparing and sequencing as samples (e.g., the same cycles of PCR). Reads that are not wild type should be counted as mistakes and the frequency of these reported.

3. The mean count of 1aa change variants is about 2000 and the mean count of 2aa change variants is about 30. This should be discussed. Is it possible that misreads could contribute to this observation. The authors should discuss barcoding strategies that can be utilized to reduce the impact of misreads. For example, see Nature Methods 2010 7:119-122. The authors should describe why they chose the direct sequencing approach that the used compared to barcoding strategies. The authors should provide an experimental analyses of misreads (see point 2 above).

4. According to the mutational strategy, the authors expected about a 1:2:2 ratio of wild-type : single mutation : double mutation. The data report about 1000 single mutations and 50,000 double

mutations. The authors should comment on this discrepancy and the potential reasons for it.

5. The estimated errors (sigma in Sup. Table 3) seem remarkably small given the read depth. Mean reads of ~10 yield errors of ~0.05 in toxicity. The authors should examine and report if the calculated errors account for the variation between experimental replicates.

Minor concerns:
1. p. 4 – T"he patterns of genetic interactions in double mutants in this region reveal that this 'unstructured' region is actually structured in vivo"

2. p. 8 – "We have shown recently that the pattern of genetic (epistatic) interactions between mutations in a protein can report on the secondary structure of that molecule when it is 8 performing the function that is being selected for(51)."

3. P. 16 – "Reads that contained base calls with Phred scores below 30 (290-331 DMS library) or below 25 (332-373 DMS library) were discarded." What was the reasoning for selecting these cutoffs – why do they differ for each library?

4. The average toxicity of stop codons appears to be about -0.15. Some discussion of what this means relative to the observed toxicity would be helpful.

5. Supplementary Fig 2D seems very interesting – as it gets at the potential disease causing properties of mutations. The authors should consider moving this to the main text.

We thank the referees for their enthusiasm and suggestions. Please see below for the responses to each point.

Reviewers' comments:

Reviewer #1 (Remarks to the Author):

In this manuscript Bolognesi, Faure, et al. use deep mutational scanning to study the impact of protein aggregation on cellular toxicity. They introduce more than fifty thousand mutations in the prion-like domain (PrD) of the intrinsically disordered human protein TDP-43 and quantified their cytotoxicity in yeast. They find that mutations increasing hydrophobicity and aggregation tend to reduce toxicity, consistent with models suggesting that mature protein aggregates are not themselves toxic. The authors propose a mechanism in which larger, solid protein aggregates titrate protein away from smaller and dynamic liquid-like condensates in the nuclear periphery. Presumably, these liquid-like protein condensates may interfere with protein transport across the nuclear membrane. The mutations with the largest effect on toxicity tend to cluster in a central hotspot withinin the PrD. The authors developed a clever computational method based on epistatic interactions that points to the in vivo secondary structure of the protein (Nature Genetics, in press) and used it in this study to show that this central hotspot, rather than being disordered, is indeed structured inside yeast cells.

This paper adds significant evidence to the body of work suggesting that protein aggregates associated with neurodegenerative diseases, in contrast to prevailing opinion, may ameliorate rather than enhance cell toxicity. This is a solid piece of work. The results are very interesting and convincing and, in my view the claims made are supported by the evidence. In my view it should be published without delay.

Minor comments:

1. Some additional introduction of the concept of liquid demixing would be helpful for many readers.

We have added the following text:
"For many proteins, aggregation depends critically on intrinsically disordered regions with a low sequence complexity resembling that of infectious yeast prions. These prion-like domains (PRDs) are also enriched in proteins that can form liquid-like cellular condensates[20–22] through liquid-demixing. This is a concentration-dependent process through which proteins can separate into two coexisting liquid phases and it has been extensively characterized both in vitro and in the cytoplasm[23]. In several proteins PRDs are necessary and sufficient for liquid-demixing demixing[23,24]. At least in vitro, insoluble aggregates can nucleate from more liquid phases[24–26], leading to the suggestion that liquid de-mixed states can mature into pathological aggregates[19]."

2. It seems to me that dosage-sensitive proteins may also lead to a fitness cost when concentration decreases below threshold. Could the authors comment?

Correct. We have changed the text to clarify we are specifically referring to proteins that are toxic when their expression is increased: "Disordered regions and low complexity sequences are also enriched in dosage-sensitive proteins that are toxic when their concentration is increased."

3. Why do the non-toxic protein variants had higher concentration as quantified by Western?

This observation is also supported by the measurements of fluorescence intensity in figure 3F where the cytoplasmic foci far from the nucleus observed for non-toxic variants have brighter fluorescence than foci at the nuclear periphery observed in toxic variants. We can speculate that this is due to the non-toxic variants being less prone to degradation, possibly because they form more solid aggregates which cannot easily be tackled by the degradation machinery. Alternatively, cells expressing toxic variants are slow-growing and this may result in reduced production of TDP-43.

4. How can we know that aggregation potential accounts for 4% of the variance on relative toxicity in the hotspot from Fig. 2F,G? Is this simply because of the R? It would be good to add a sentence or two explaining this.

The proportion of toxicity variance explained by hydrophobicity in the hotspot is R-squared = (-0.81)^2 = 66%. We controlled for the effect of hydrophobicity by taking the residuals from a linear regression of toxicity on hydrophobicity, were we restricted the analysis to variants in the hotspot. Next, we calculated the proportion of variance in these residuals explained by aggregation potential i.e. R-squared = (-0.65)^2 = 12%. Because the residual toxicity variance (after controlling for hydrophobicity) is only 100-66 = 34% of the total toxicity variance (before controlling for hydrophobicity), we conclude that aggregation potential accounts for an additional 0.12*0.34 = ~4% of toxicity variance in the hotspot. This is explained in the legend of Fig. 2.

5. In my view Panel 2C does not show that "None of them are as predictive as hydrophobicity"

This has now been corrected as a reference to 2B where the different predictors are shown.

Reviewer #2 (Remarks to the Author):

To explore the relationship between TDP-43 aggregation and toxicity, here the authors used deep mutagenesis, generating >50,000 mutations comprising of 1266 single and 56, 730 double mutants, in the PRD of TDP-43 and quantifying toxicity in yeast. Of these mutants, 18032 were more toxic than WT TDP-43 and 16152 less toxic. The authors found that mutations that increased the aggregation propensity of TDP-43 correlated with reduced toxicity, whereas those that promoted dynamic, liquid condensates correlated with increased toxicity. Interestingly, increasing the hydrophobicity of TDP-43, which is associated with reduced toxicity, caused a relocalization of the protein away from the nuclear membrane and formation of larger aggregates. Mutational effect was strongest in a 31 aa hotspot at residues 312-342 with mutations to charged or polar residues increasing toxicity and to hydrophobic aa decreasing toxicity. From these findings, the authors propose that TDP-43 aggregation is neuroprotective by titrating from liquid-like states.

This is an interesting observation. Comments are below

1. What was the finding from disease causing mutations e.g. A315T, M337V etc ?

This is indeed an important question. As stated in the original text, TDP-43 mutations reported in ALS increase toxicity, with a strong bias towards moderate effects (Fig. 1D, Supplementary Figure 2D). In the revised manuscript, we also characterise the localisation of four ALS variants (3 sporadic, 1 familial). These variants also have an increased number of small foci at the nuclear periphery, albeit less so than the most toxic variants in our assay (Supplementary Figure 6).

2. WT TDP-43 should be included in Fig 3A

Images for YFP-tagged WT TDP-43 are included in Fig 3A.

3. Further characterization of the different types of TDP-43 inclusions is required e.g. are the aggregates p62 positive, do the droplets co-localize with stress granule markers, nuclear pore proteins?

We tested for co-localization of toxic and non-toxic TDP-43 variants with the 10 proteins listed in the table below in order to understand if the different foci we observe correspond to previously described compartments and if toxic variants can specifically interact with nuclear pore proteins. Co-localization was only observed for both types of TDP-43 variants with the Hsp104 chaperone. The results of these additional experiments are reported in Fig. S9 and referred to from the Results.

Protein	***Localization/ Reasons for testing***	***Reference***	***Co-localization with TDP variants***
Rnq1	***Ipod***	***Kaganovich 2008***	***no***

Hsp104	***Junq / Ipod***	***Kaganovich 2008***	***yes***
Hsp42	***Ipod***	***Hill 2017***	***no***
Tho2	***Mislocalized when TDP-43 forms foci in HEK293T cells upon stress***	***Woerner 2016***	***no***
Rkr1 (ltn1)	***Ipod***	***Hill 2017***	***no***
Sti1	***Regulates Junq formation***	***Hill 2017***	***no***
Nsp1 (nup62)	***Co-localizes with cytoplasmic TDP-43 foci in neurons upon stress***	***Gasset-Rosa 2019***	***no***
Gsp1 (ran)	***Mislocalized when TDP-43 forms foci in neurons upon stress***	***Gasset-Rosa 2019***	***no***
Srp1 (importin alpha)	***Co-localizes with cytoplasmic TDP-43 foci in neurons upon stress***	***Gasset-Rosa 2019***	***no***
Nup145 (nup98)	***Co-localizes with WT TDP 43 in N2a cells***	***Chou 2018***	***no***

Reviewer #3 (Remarks to the Author):

The manuscript by Bolognesi et al. describes deep mutational scanning results of TDP-43 toxicity in yeast. This review is focused on the deep mutational scanning approach and data. The authors purchased about 150 based synthetic oligos doped with random mutations at a frequency intended to yield a ratio of about 1:2:2 of WT: single mutation: double mutation. 2 libraries of mutants were cloned into galactose inducible plasmids, introduced into budding yeast, and subject to bulk growth competitions under induced conditions. The frequency of each mutant was estimated by paired-end sequencing of samples prior to and after competition. The change in frequency of each mutated variant was used to estimate impacts on growth rate.

Many aspects of the mutational scanning data appear to have been done with careful thought. However, there are also aspects of the mutational scanning data that are not clearly described or that may be potentially misleading and should be addressed prior to publication. The

correlation between replicates (Fig 1B) is impressive and indicates that stochastic noise to signal is favorable.

Major concerns:

1. The treatment of biological replicates is unclear. Four biological replicates are mentioned in the method section, but two replicates are presented in Fig 1B, and one set of measurements is provided in Sup. Table S3. The authors need to clarify how replicates were performed and analyzed. Each replicate needs to be reported in a separate Supplementary Table. This is imperative not only for judging the merit of the data, but also for potential future analyses to distinguish biological from technical variation.

Four independent replicate experiments (starting from independent yeast transformations) were performed for each library. The input counts of all variants in all replicates are compared in Supplementary figure 1 A and B. For both libraries, before downstream analysis, one replicate was excluded on the basis of considerably lower correlation of variant input counts with the other replicates, as highlighted in Supplementary figure 1 A and B. This is stated in the Methods (Sequencing data pre-processing). The toxicity scores for all single and double mutants in the remaining three replicates are compared in Supplementary figure 1 D and E. All figures in the paper rely on toxicity estimates corresponding to the error-weighted mean across the remaining three replicate selections (Variant toxicity and Error Estimates). We have added the replicate toxicity scores to Supplementary Table 3 as requested.

2. Misreads need to be experimentally examined. Assuming that paired-end reads eliminate mis-reads is unwise. Potential misreads should be assessed using a homogenous starting sequence (e.g. wild-type plasmid) and the same procedure for preparing and sequencing as samples (e.g., the same cycles of PCR). Reads that are not wild type should be counted as mistakes and the frequency of these reported.

As in Diss & Lehner, 2018, we examined misreads (sequencing errors) by measuring the per base error probability in the 'constant' regions, i.e. 10bp upstream and 10bp downstream of the 'variable' (doped) regions of TDP-43, that were used as primer annealing sites for library amplification. The sequences in the constant regions should be identical to the wild type and any differences are likely sequencing errors. We estimate the frequency of an incorrect base call to be 0.0001 (sd = 6e-5) and 0.0004 (sd = 4e-4) for the 290-331 and 332-373 DMS libraries respectively. Differences in error frequencies between the two DMS libraries are most likely caused by different Phred cutoffs used (see reply to Minor Concern 3), but little variability is observed in error frequencies between individual sequencing libraries (3 input replicates and 2x3 output replicates) from the two DMS libraries. Moreover there is little variability between error probabilities depending on the wild-type base ([4e-5,2e-4] for the 290-331 DMS library and [1e-4,1.5e-3] for the 332-373 DMS library). This is now presented in the methods (Variant toxicity and error estimates)

3. The mean count of 1aa change variants is about 2000 and the mean count of 2aa change variants is about 30. This should be discussed. Is it possible that misreads could contribute to this observation. The authors should discuss barcoding strategies that can be utilized to reduce the impact of misreads. For example, see Nature Methods 2010 7:119-122. The authors should describe why they chose the direct sequencing approach that the used compared to barcoding strategies. The authors should provide an experimental analyses of misreads (see point 2 above).

The difference in counts for single and double AA variants is to be expected (based on the random mutagenesis) and is related to the difference in the total number of possible variants of each type i.e. 1,596 single and 621,642 double mutants respectively.

With a per base misread frequency of 0.0001 (as derived above), the probability of observing at least one base call error per read is 126bp*0.0001 = 0.0126. The total number of reads associated with single nucleotide substitution variants is ~2e6 for a typical input sample from the 290-331 DMS library. However, we estimate that these observed single nucleotide variants represent only 1-0.0126 = 98.74% of 'true' single nucleotide variants. Therefore we expect an additional 0.0126/0.9874*2e6 = 2.6e4 'misread' counts originating from single nucleotide variants.

For the sake of this example we will assume that all misreads originating from single nucleotide variants were attributed (mistakenly) to double nucleotide variants. Indeed, the most likely origin of 'misread' double nucleotide variants is a single nucleotide variant with an additional misread base.

For a nucleotide sequence of length 126bp (doped region) there are (126 choose 2) = 7875 possible pairs of base positions that can each be mutated to one of three possible nucleotides. Therefore there exist a total of 7875*3*3 = ~7e4 possible double nucleotide variants. Even in the scenario where all counts from 'misread' single nucleotide variants are distributed equally among all possible double nucleotide variants, we expect only 2.6e4/7e4 = ~0.5 an additional count per double nucleotide variant attributable to sequencing errors (i.e. ~1% based on the observed mean count for double nucleotide variants of ~37). Even considering the higher misread frequency in the 332-383 DMS library (0.0004), we still expect <2 additional counts per double nucleotide variant attributable to sequencing errors.

These calculations, which we have added to the methods (Variant toxicity and error estimates), demonstrate that double AA variant counts are not dominated by misreads. However, we agree with this reviewer that barcoding strategies could further reduce the impact of misreads. We now mention this potential improvement in the Methods .

4. According to the mutational strategy, the authors expected about a 1:2:2 ratio of wild-type : single mutation : double mutation. The data report about 1000 single mutations and

50,000 double mutations. The authors should comment on this discrepancy and the potential reasons for it.

As mentioned in a related comment above, the observed difference in the total number of unique single and double AA variants is to be expected (based on the random mutagenesis) and is related to the difference in the total number of possible variants of each type i.e. 1,596 and 621,642 respectively.

5. The estimated errors (sigma in Sup. Table 3) seem remarkably small given the read depth. Mean reads of ~10 yield errors of ~0.05 in toxicity. The authors should examine and report if the calculated errors account for the variation between experimental replicates.

a) ***Indeed, the Poissonian error for a toxicity estimate derived from ~10 reads would be ~33% relative error (error ~ 1/sqrt(10 reads)). However, the toxicity estimates are subsequently normalized for #generations of the selection experiment (on average ~6 generations) and merged across replicate selections (in ideal cases leading to a 1/sqrt(3) reduction of errors). This therefore explains the ~0.05 errors in reported toxicity estimates of low count variants. (note that reported errors are absolute errors on natural logarithm scale that toxicity estimates are reported in)***
b) ***The calculated errors do indeed reflect the variation between experimental replicates. As explained in the Methods (Variant toxicity and error estimates Section), under minimal assumptions, errors are a combination of Poissonian errors due to finite sequencing counts and variability between replicates, with variances of these two processes being additive, i.e.*** $\epsilon_i^2 = \epsilon_{counts,i}^2 + \epsilon_{rep}^2$ ***as the error for variant i. Here,*** $\epsilon_{counts,i}^2$ ***depends on the number of sequencing counts for variant i (and the wild-type variant used for normalization) in input and output replicate, which therefore increases for low count variants.*** ϵ_{rep}^2***, the variability added due to differences between replicate selections, instead is a constant and the same for all variants. Consistently, we find that variants with high variation between replicates (y-axis in figure below) are well explained by purely count-based error estimates (x-axis in figure below), while variants with low variation between replicates approach a lower bound of variation, the replicate error*** ϵ_{rep}^2***. Indeed, a nonparametric estimate of the average of y given x (red line) closely follows the expectation given the full error formula (blue line, i.e. replicate + count based error). Note that the variability in standard deviation of toxicity estimates of individual variants having the same count-based error estimates inadvertently arises from the low number (3) of replicates used to estimate the standard deviation.***

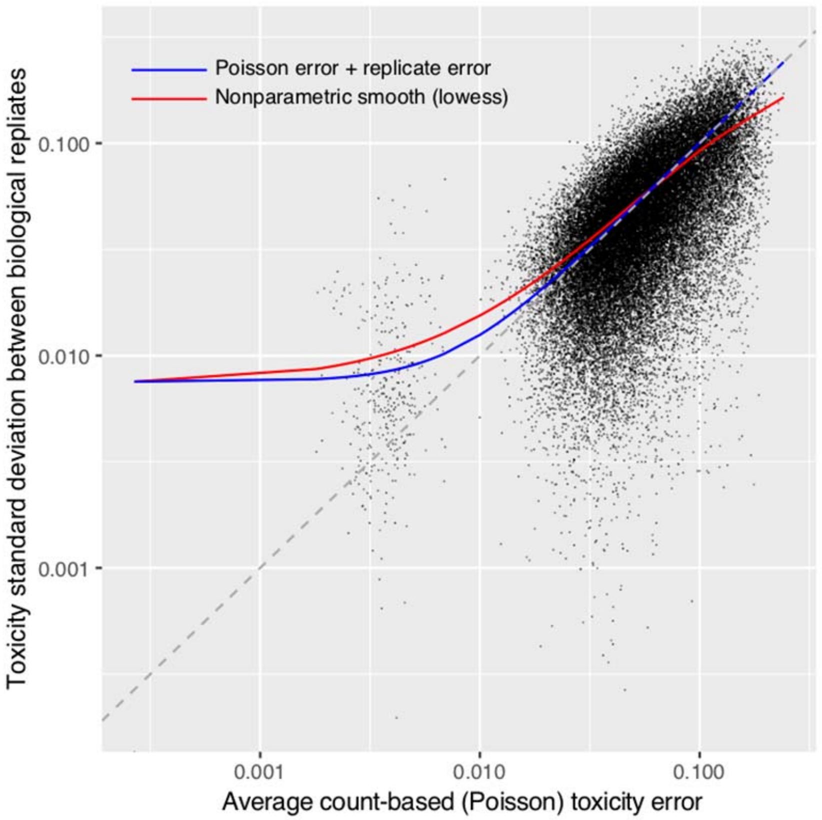

Per variant comparison of purely count-based toxicity error estimates (average across counts in three biological replicates) versus standard deviation of toxicity estimates across three biological replicates in the 290-332 DMS library.

Minor concerns:
1. p. 4 – T"he patterns of genetic interactions in double mutants in this region reveal that this 'unstructured' region is actually structured in vivo"

No suggested edit noted. Please clarify the suggestion.

2. p. 8 – "We have shown recently that the pattern of genetic (epistatic) interactions between mutations in a protein can report on the secondary structure of that molecule when it is 8 performing the function that is being selected for(51)."

No suggested edit noted. Please clarify the suggestion.

3. P. 16 – "Reads that contained base calls with Phred scores below 30 (290-331 DMS library) or below 25 (332-373 DMS library) were discarded." What was the reasoning for selecting these cutoffs – why do they differ for each library?

The quality scores of reads in the 332-373 library were overall slightly lower than in the 290-331 library. In order to retain comparable numbers of variants in both libraries, we chose a more lenient Phred score threshold for the former. The chosen thresholds

explain the observed frequency of incorrect base calls (see above): Phred 30 -> error probability 10^{-3}; Phred 25 -> error probability $10^{-2.5}$ ~ 0.003

4. The average toxicity of stop codons appears to be about -0.15. Some discussion of what this means relative to the observed toxicity would be helpful.

In our selection experiment cells expressing stop codon variants grow faster than cells expressing WT TDP-43, with a very similar effect to non-toxic variants (as highlighted in Fig. 1D). This means the introduction of stop-codons in this region relieves almost all the toxicity induced by expression of WT TDP-43, consistent with previous observations by Johnson et al. (PNAS, 2008) showing no toxicity to yeast cells upon expression of residues 1-306 of TDP-43.

5. Supplementary Fig 2D seems very interesting – as it gets at the potential disease causing properties of mutations. The authors should consider moving this to the main text.

We have now added a boxplot depicting the toxicity distribution of human disease mutations to Fig. 1D.

REVIEWERS' COMMENTS:

Reviewer #1 (Remarks to the Author):

In my view the authors have done an excellent job responding to my comments and those of the other reviewers. I recommend that this exciting work be published without delay.

Reviewer #3 (Remarks to the Author):

The authors have addressed my concerns.
